# Targeting CD22 with the monoclonal antibody epratuzumab modulates human B-cell maturation and cytokine production in response to Toll-like receptor 7 (TLR7) and B-cell receptor (BCR) signaling

**DOI:** 10.1186/s13075-017-1284-2

**Published:** 2017-05-15

**Authors:** Natalia V. Giltiay, Geraldine L. Shu, Anthony Shock, Edward A. Clark

**Affiliations:** 1 Division of Rheumatology, Department of Medicine, University of Washington, Seattle, WA 98109 USA; 2 Department of Immunology, University of Washington, Seattle, WA 98109 USA; 3 UCB Celltech, Slough, UK

**Keywords:** Antibodies, B cells, B-cell targeted therapy, CD22, IL-6, IL-10, Epratuzumab, Systemic lupus erythematosus, TLR7

## Abstract

**Background:**

Abnormal B-cell activation is implicated in the pathogenesis of autoimmune diseases, including systemic lupus erythematosus (SLE). The B-cell surface molecule CD22, which regulates activation through the B-cell receptor (BCR), is a potential target for inhibiting pathogenic B cells; however, the regulatory functions of CD22 remain poorly understood. In this study, we determined how targeting of CD22 with epratuzumab (Emab), a humanized anti-CD22 IgG1 monoclonal antibody, affects the activation of human B-cell subsets in response to Toll-like receptor 7 (TLR7) and BCR engagement.

**Methods:**

B-cell subsets were isolated from human tonsils and stimulated with F(ab′)_2_ anti-human IgM and/or the TLR7 agonist R848 in the presence of Emab or a human IgG1 isotype control. Changes in mRNA levels of genes associated with B-cell activation and differentiation were analyzed by quantitative PCR. Cytokine production was measured by ELISA. Cell proliferation, survival, and differentiation were assessed by flow cytometry.

**Results:**

Pretreatment of phenotypically naïve CD19^+^CD10^–^CD27^–^ cells with Emab led to a significant increase in IL-10 expression, and in some but not all patient samples to a reduction of IL-6 production in response to TLR7 stimulation alone or in combination with anti-IgM. Emab selectively inhibited the expression of *PRDM1*, the gene encoding B-lymphocyte-induced maturation protein 1 (Blimp-1) in activated CD10^–^CD27^–^ B cells. CD10^–^CD27^–^IgD^–^ cells were highly responsive to stimulation through TLR7 as evidenced by the appearance of blasting CD27^hi^CD38^hi^ cells. Emab significantly inhibited the activation and differentiation of CD10^–^CD27^–^IgD^–^ B cells into plasma cells.

**Conclusions:**

Emab can both regulate cytokine expression and block Blimp1-dependent B-cell differentiation, although the effects of Emab may depend on the stage of B-cell development or activation. In addition, Emab inhibits the activation of CD27^–^IgD^–^ tonsillar cells, which correspond to so-called double-negative memory B cells, known to be increased in SLE patients with more active disease. These data may be relevant to the therapeutic effect of Emab in vivo via modulation of the production of pro-inflammatory and anti-inflammatory cytokines by B cells. Because Blimp-1 is required by B cells to mature into antibody-producing cells, inhibition of Blimp1 may reduce autoantibody production.

**Electronic supplementary material:**

The online version of this article (doi:10.1186/s13075-017-1284-2) contains supplementary material, which is available to authorized users.

## Background

B cells are central mediators of humoral immunity and play a key role in the protection against pathogens. However, aberrant B-cell activation is a common feature of many autoimmune diseases including systemic lupus erythematosus (SLE), primary Sjögren’s syndrome (pSS), and rheumatoid arthritis (RA). B cells may contribute to the pathogenesis of autoimmune diseases through several mechanisms, including both antibody (Ab)-dependent functions (e.g., secretion of autoantibodies (auto-Abs)) and Ab-independent functions, such as antigen (Ag) presentation to T cells and production of pro-inflammatory cytokines [1, 2].

SLE is a complex, systemic autoimmune disease with highly diverse clinical manifestations [3]. The hallmarks of the disease are polyclonal B-cell activation, production of auto-Abs against nuclear-containing Ags, and organ damage due to immune complex deposition. Abnormalities in various B-cell compartments have been described in SLE patients, including expansion of newly formed (transitional) cells, as well as an expansion of Ag-experienced CD27^+^/IgD^–^ switched memory B cells and CD27^–^IgD^–^ double-negative (DN) memory B cells [2, 4–6]. Previous studies have shown that the frequency of circulating CD20^–^CD27^++^ plasma cells correlates with SLE disease activity and auto-Ab titers [7]; however, the cellular origins of Ab-producing cells in SLE as well as the signals that drive their activation are not well defined. Alterations in the balance of signals downstream of the B-cell receptor (BCR) and pro-survival signals may contribute to the loss of immune tolerance and to the survival and activation of autoreactive B cells [8, 9]. Dysregulation of BCR signaling, including increased phosphorylation of Syk and decreased phosphorylation of phosphatase activities, has also been described in SLE patients [10, 11].

B-cell activation via innate immune receptors, including endosomal Toll-like receptors (TLRs) expressed in B cells, may play a key role in driving autoreactive B cells in SLE [9, 12–15]. In mice, RNA-associated autoantigens activate autoreactive B cells by engaging BCRs and TLR7, an endosomal TLR specialized in the recognition of viral ssRNA, and induce the production of auto-Abs [12, 16]. Data from murine lupus models further support a role for TLR7 in SLE pathogenesis. Extra copies of the *Tlr7* gene drive lupus-like disease [17–19]; whereas lupus-prone *Tlr7*-deficient mice develop attenuated disease symptoms [20, 21]. Importantly, B-cell-intrinsic TLR7 signals can drive cell proliferation and differentiation and amplify auto-Ab production to further exacerbate SLE disease in mice [22]. In humans, genetic studies have demonstrated a correlation between genetic variations associated with dysregulation of TLR7 expression and SLE susceptibility [23–25].

Despite significant evidence implicating TLR7 in SLE, the effects of TLR7 on human B cells have not been explored fully. Little is known about the signals that can regulate TLR7-mediated activation of human B cells. IFN-α can promote human B-cell responses to TLR7 ligation, most likely through its ability to upregulate TLR7 receptor levels [26]. In-vitro stimulation of CD19^+^CD27^–^ blood B cells with a synthetic TLR7 ligand induces IgM and IgG production as well as secretion of IL-6 and IL-10 [27]. TLR7 activation also expands IgM^+^CD27^+^ memory B cells and CD27^hi^ B cells, and a combination of TLR7 plus IFN-α promotes the production of auto-Abs [28]. Thus, TLR7-mediated activation of human B cells, as with mouse B cells, can induce the production of auto-Abs.

While signals from BCR and TLR7 can synergize and promote inappropriate activation of autoreactive B cells, the engagement of other surface receptors, such as CD19 and CD22, have been proposed to inhibit their activation [29, 30]. The CD22 Siglec receptor family member is expressed predominantly on B cells and binds via its extracellular ligand-binding domain to α2-6-linked sialic acids on glycoproteins expressed on the same cell (*cis* interactions) or on opposing cells and/or soluble proteins (*trans* interactions) [31, 32]. CD22 acts as an adhesion receptor and functions to regulate B-cell migration [33–35]. Crosslinking of CD22 and the BCR triggers phosphorylation of the CD22 cytoplasmic tail, leading to the activation of a number of signaling molecules, known to either inhibit the BCR signaling or to promote the activation of JNK/SAPK and mitogen activated protein kinase ERK2 [30, 36, 37]. In addition to its function in regulating BCR signaling, CD22 has been implicated in the regulation of TLR-mediated signaling in B cells [38]. CD22^–/–^ B cells have hyperactive responses to TLR stimulation compared to wild-type (WT) B cells [38, 39]. Furthermore, studies have shown that LPS-induced activation of nuclear factor-κB (NF-κB) downstream of TLR4 is inhibited by the expression of CD22 [38].

The expression of both CD22 and its ligands vary according to the B-cell maturation/activation state. In the periphery, CD22 is expressed at maximum density on human CD27^–^ naïve and transitional B cells, while it is downregulated by plasma cells [40, 41]. CD22 availability on the cell surface is also dependent on masking or unmasking of CD22 by endogenous (*cis*) ligand interactions [42]. The expression of CD22 ligands on human B cells is less well explored, but recent studies have shown that, due to changes in glycosylation, germinal center (GC) B cells lose the expression of high-affinity CD22 ligands, leading to CD22 “unmasking” [43].

The development of monoclonal Abs designed to target human CD22 [44] as well recent advances in human B-cell phenotyping [45] provide new opportunities to explore the effects of CD22 engagement on different subsets of human B-cell subsets. Epratuzumab (Emab), a humanized anti-human CD22 IgG1 Ab, has previously shown promising clinical activity both as a single agent and in combination with rituximab in patients with non-Hodgkin’s lymphoma (NHL) [46]. Unlike rituximab, which depletes circulating B cells, Emab does not induce complement-dependent cytotoxicity or Ab-dependent cellular cytotoxicity [47]. CD22 ligation by Emab, however, provokes rapid internalization and phosphorylation of CD22, inhibits the phosphorylation of Syk and PLCγ2, and reduces intracellular Ca^2+^ mobilization after BCR stimulation in vitro [44, 48, 49]. Given the role of CD22 in modulating both BCR and TLR signaling, targeting CD22 with Emab has also been explored as a therapy for autoimmune diseases, including SLE and pSS [50, 51]. Phase I and IIb clinical trials have demonstrated clinically relevant, sustained improvements in patients with moderate-to-severe SLE and a good safety profile of Emab [52, 53]. Emab treatment was found to induce a partial reduction of circulating B cells in SLE patients affecting primarily CD27^–^ cells [54], a phenomenon that later led to an exploration of the in-vitro effects of Emab on the expression of the adhesion molecules CD62L, β7 integrin, and β1 integrin and on B-cell migration toward CXCL12 [41]. CD22 binding by Emab also induces a reduction of CD19, CD21, and CD79b expression through a process known as trogocytosis (i.e., Fc-mediated receptor “shaving” to other effector cells). In line with these findings, a decrease in CD19 expression was observed in SLE patients treated with Emab [55]. Recent studies have also investigated the effects of Emab on cytokine production. Emab inhibited IL-6 and tumor necrosis factor alpha (TNF-α) production of blood B cells isolated from healthy donors and SLE patients in response to BCR crosslinking alone or in combination with TLR9 ligand CpG [56]. Overall, the available data led to the hypothesis that the primary mode of action of Emab is to enhance the normal inhibitory role of CD22 on B-cell activation [57].

Given the importance of TLR7 signaling in activating autoreactive B cells in SLE, we investigated whether CD22 crosslinking by Emab might affect B-cell activation in response to BCR and/or TLR7 stimulation. We further aimed to identify which subpopulation of human B cells might be affected to the greatest extent by Emab in the context of BCR/TLR7 stimulation. Using human tonsillar CD10^–^CD27^–^ B cells, we found that Emab modulates cytokine production by inhibiting IL-6, while at the same time enhancing IL-10 production. Emab dramatically and selectively inhibited levels of *PRDM1*, the gene encoding Blimp-1 in CD10^–^CD27^–^ tonsillar B cells, activated by either TLR7 signaling alone or in combination with BCR stimulation. Tonsillar B-cell subsets responded differently to BCR/TLR7 stimulation; among them CD10^–^CD27^–^IgD^–^ cells, which correspond to DN memory B cells found in the periphery, were most responsive to TLR7 stimulation, as evidenced by the appearance of CD27^hi^CD38^hi^Blimp1^+^ plasmablasts. In particular, Emab inhibited the in-vitro differentiation of CD10^–^CD27^–^IgD^–^ and CD10^+^CD27^–/+^ B cells and reduced the survival of CD10^+^CD27^–/+^ B cells but not other B-cell subsets. Thus, Emab can both enhance and inhibit TLR7-driven B-cell responses: depending on the B-cell developmental/maturation state, CD22 crosslinking by Emab affects B-cell survival, activation, and differentiation differently, which may have important implications for the clinical use of Emab and monitoring of CD22-based therapies.

## Methods

### Cell preparation and purification

Post-surgical tonsillar tissue samples were obtained from Valley Medical Day Surgery Center (Renton, WA, USA) in accordance with an IRB approved protocol. Cell suspensions were prepared by gently teasing tissues in R10 medium (RPMI 1640 containing 10% FBS (ThermoFisher Scientific), 100 U/ml penicillin, 100 mg/ml streptomycin, 2 mM l-glutamine, 1 mM sodium pyruvate, and 10 mM Hepes) with forceps and scissors, and then separating cells over Ficoll-Hypaque (GE Healthcare Life Sciences). Tonsillar B cells were enriched by depleting CD2^+^ T and NK cells using sheep erythrocytes, a technique that relies on the formation of immunorosettes (i.e., rosetting) [58], and then separated again over Ficoll-Hypaque. Samples after rosetting were typically ≥90% pure CD20^+^ B cells. In some experiments, cells were further enriched for CD27^–^CD10^–^ B cells using biotinylated monoclonal Abs (mAbs) against CD3 (G19-4), CD5 (10.2), CD10 (CB-CALLA), and CD27 (O323) (eBioscience) with the StemCell Technologies Human Biotin Selection Kit for negative selection. In other experiments, post-rosetted cells were stained with fluorescently labeled mAbs (anti-CD3, CD27, CD10, and IgD antibodies) and then sorted into CD10^–^CD27^+^ (memory), CD10^–^CD27^–^IgD^+^ (naïve), and CD10^–^CD27^–^IgD^–^ (IgD^–^CD27^–^ DN memory) B-cell and CD10^–^CD27^+/–^ (pre-GC/CG/plasma) cell populations using a FACSAria II high-speed cell sorter (BD Pharmingen) at 4 °C under sterile conditions. Post-sort analyses were performed to assess the purity of sorted cells. Peripheral blood mononuclear cells (PBMCs) from healthy donors (HD) were isolated by density-gradient centrifugation using Ficoll-Hypaque. Written consent was obtained from all blood donors.

### In-vitro cell culture and CFSE proliferation assay

B cells were cultured in R10 medium at 37 °C and 5% CO_2._ For gene expression analyses, cells were enriched for CD27^–^CD10^–^ B cells and plated at 3 × 10^6^ cells per ml with preincubation for 1 hour at 37 °C with Emab (5 μg/ml) or hIgG1 isotype control (5 μg/ml; Sigma) or R10 medium and then stimulated with TLR7 agonist R848 (50 ng/ml; InvivoGen), F(ab′)_2_ anti-human IgM (5 μg/ml; Jackson ImmunoResearch Laboratories, Inc.), or a combination of R848 plus anti-IgM. In some cases, cells were pretreated with IFN-α at 1000 U/ml (PBL, Piscataway, NJ, USA). Samples were cultured for 12 hours, harvested, and used for RNA isolation. For cell proliferation experiments, cells were loaded with 2.5 μM CFSE (ThermoFisher Scientific) in PBS at 37 °C for 10 min, quenched with R10 medium, washed, stimulated, and then cultured for 3 days. For assessing cell survival and plasma cell differentiation, sorted B-cell subsets were placed in 96-well plates at a concentration of 2.5 × 10^6^ cells per ml, treated with different stimuli, and analyzed by flow cytometry after 3–5 days of in-vitro cell culture.

### Cytokine production

B cells enriched for CD10^–^CD27^–^ cells were plated at 5 × 10^6^ cells per ml in 96-well plates, preincubated with or without Emab or a human IgG control, and stimulated with R848 and/or F(ab′)_2_ anti-human IgM. Culture supernatants were collected 3 days post stimulation, frozen down, and used to assess cytokine production. Cytokine array (R&D Systems) data showed significant induction of IL-6 and IL-10 after R848 and/or anti-IgM stimulation. Further quantification of these cytokines was performed using Human Quantikine ELISA Kits (R&D Systems) according to the manufacturer’s instructions.

### Flow cytometry

For characterization of tonsillar B-cell subsets, single cell suspensions were stained with appropriate combinations of fluorescently labeled mAbs, including anti-CD10 (CB-CALLA), CD19 (SJ25C1), CD20 (2H7), CD22 (4KB128 and S-HCL-1), CD27 (LG.7F9), CD38 (HB7) (eBioscience), IgD (IA6-2), CD3 (SP34-2), and CD95 (DX2) (BD Biosciences). Live cells were identified using LIVE/DEAD Fixable Near-IR staining (Molecular Probes) according to the manufacturer’s instructions. Cultured cells were pelleted, washed, and stained with LIVE/DEAD, followed by surface staining with fluorescently labeled mAbs. For Blimp1 intracellular staining, cells were first stained with LIVE/DEAD fixable dye, washed, stained with appropriate surface markers, washed and then fixed, permeabilized, and stained with PE-conjugated rat IgG2a*k* anti-Blimp1 Ab (6D3) using the Transcription Factor Buffer Set (BD). CFSE-labeled cells were cultured for 3 days and the levels of cell proliferation were measured based on CFSE dilution. Multicolor flow cytometry was performed using a five-laser LSRII flow cytometer (BD) and analyzed with FlowJo software (Tree Star).

### Imaging flow cytometry

Emab anti-CD22 binding and internalization by tonsillar B cells was assessed by multispectral imaging flow cytometry. Tonsillar B cells were stained with mAb specific for CD10, CD20, CD27, and IgD with or without Emab, conjugated to Pacific Blue (conjugation was performed using Pacific Blue™ Antibody Labeling Kit from Molecular Probes, ThermoFisher Scientific). Incubation with Pacific Blue-Emab was performed at either 4 °C on ice in the presence of NaN_3_ or at 37 °C for 30 min. CD20^+^ cells were gated into CD10^–^CD27^–^, CD10^–^CD27^+^, and CD10^–^CD27^+/–^ B-cell subsets, and Emab binding and receptor-mediated internalization was determined for each subset. Then 50,000–100,000 cells were analyzed using 60× camera magnification using an Image Stream X Mark II instrument and data were analyzed with IDEAS software (Amnis). The Internalization Score (IS) was defined as the ratio of intensity inside the cell to the intensity of the entire cell.

### Quantitative RT-PCR

Total RNA was extracted from cells using an RNeasy mini kit with DNase treatment (QIAGEN). First-strand cDNA was generated using 250 ng of total RNA with the SuperScript III high-capacity cDNA RT-kit using random primers (Invitrogen). Primers, as indicated in Additional file 1: Table S1, were synthesized (Invitrogen) and diluted to the appropriate concentrations using molecular-grade water. Transcript expression was analyzed by quantitative RT-PCR using SYBR® green PCR Master Mix (Applied Biosystems) on an Applied Biosystems StepOnePlus Real Time PCR System using a two-stage cycle of 95 °C for 15 s and 60 °C for 1 min repeated for 40 cycles, followed by a dissociation stage. Threshold cycle (Ct) values were determined by setting a constant threshold at 0.2. All samples were normalized for the expression of 18S; fold changes in gene expression were calculated using the 2^−ΔΔCT^ method and presented as relative expression to unstimulated controls.

### Statistical analyses

Graphs and statistical analyses were performed using Prism 5.0 software (GraphPad, San Diego, CA, USA). Statistical significance between groups was determined by two-tailed, unpaired Student’s *t* test or by one-way ANOVA with Turkey post test. Pearson’s correlation was used to measure the relationship between two variables. Results are reported as mean ± SD or ± SEM. *p* < 0.05 was considered statistically significant.

## Results

### CD22 is expressed broadly across tonsillar B-cell subpopulations and is internalized in response to Emab

**Fig. 1:**
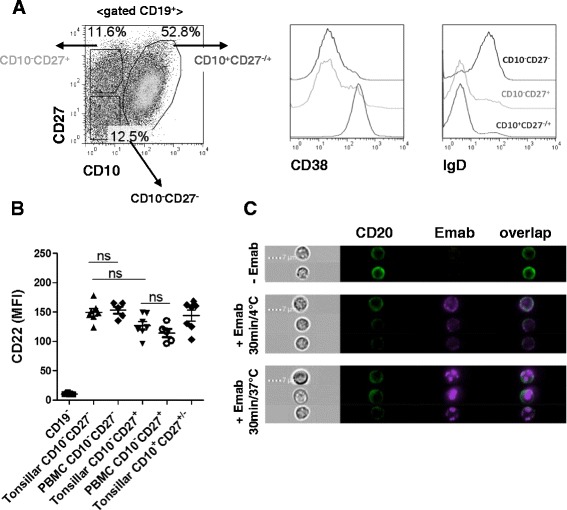
CD27^–^CD10^–^ tonsillar B cells express CD22 and internalize Emab. **a**, **b** Characterization of tonsillar B-cell subsets. Tonsillar cells were stained with fluorochrome-labeled mAbs against CD10, CD19, CD22, CD27, CD38, and IgD and analyzed using multicolor flow cytometry. **a** Representative flow analysis of gated live CD19^+^ cells showing B-cell subgating into CD10^–^CD27^–^, CD10^–^CD27^+^, and CD10^+^CD27^+/–^ subsets; *right* histograms show expression of CD38 and IgD within each population. **b** Surface expression of CD22 quantified by flow cytometry and compared to corresponding B-cell subsets from PBMCs of healthy donors. Each *dot* on the graph represents individual donors. **c** Tonsillar B cells were stained with mAb specific for CD10, CD27, and CD20 (*green*) and with Emab, conjugated to Pacific Blue (*purple*). Cells were incubated at 4 or 37 °C, and Emab surface binding and internalization of CD10^–^CD27^–^ cells was visualized by imaging flow cytometry. Data shown are representative of three independent experiments. *Emab* epratuzumab, *PBMC* peripheral blood mononuclear cell

Human tonsils contain 50–60% CD19^+^ cells and thus represent a useful source of B cells, including cells with various phenotypes and activation states. Previous analyses showed that 60–80% of purified dense tonsillar B cells are CD22^+^, including both IgD^+^ and IgD^–^ cells [59]. To determine the levels of CD22 expression on tonsillar B-cell subsets in more detail, we performed a multicolor flow cytometry analysis, using mAbs against CD19, CD10, CD27, IgD, CD38, and CD22. As Fig. 1a shows, CD19^+^ tonsillar B cells can be separated into three distinct populations based on their relative expression of CD10 and CD27: first, CD10^–^CD27^–^ cells, which consist mostly of naïve B cells that are also CD38^lo^ and IgD^+^; and, second, a CD10^–^CD27^+^ cell population comprised of memory B cells, including IgD^–^ (switched) and IgD^+^ (unswitched) CD27^+^ cells. Counterparts of these two populations are found within the PBMC pool. A third, highly abundant population of B cells found in the tonsils but not in the blood is CD10^+^CD27^–/+^CD38^+^, a heterogeneous population including pre-GC, GC, and post-GC (plasmablast/plasma cell) B cells [60, 61]. Surface staining with CD22 at 4 °C (to prevent receptor internalization) showed that CD22 was expressed on all B-cell subsets but that the CD10^–^CD27^–^ B cells expressed higher levels of CD22 (average MFI 148) than CD10^–^CD27^+^ memory B cells (MFI 120) or CD10^–^CD27^–/+^ (GC/plasma cell-associated) B cells (MFI 130) (Fig. 1b); however, these differences were not statistically significant. We also compared CD22 expression levels on CD19^+^CD10^–^CD27^–^ and CD10^–^CD27^+^ tonsillar B cells to those of CD19^+^CD10^–^CD27^–^ and CD10^–^CD27^+^ blood B cells from healthy donors and found no significant differences.

Using imaging flow cytometry, we found that CD10^–^CD27^–^ cells very efficiently and uniformly internalized Emab (IS between 0.8 and 0.9) (Fig. 1c). Consistent with CD22 expression data, a significant percentage of cells within the other two subsets (e.g., CD10^–^CD27^+^ and CD10^+^CD27^–/+^ cells) also internalized Emab (data not shown). Thus, human tonsils can be used to investigate the differential effects of CD22 crosslinking by Emab on the responses of B-cell subsets from human lymphoid tissue. In particular, the CD19^+^CD10^–^CD27^–^ B cells express high levels of CD22 and efficiently internalized Emab. Because this population likely corresponds to circulating CD27^–^ B cells that are significantly decreased upon Emab administration in vivo [54], we focused our study initially on this B-cell subset. We developed a protocol for enrichment of CD10^–^CD27^–^ B cells using a two-step enrichment process, including rosetting and depletion of CD3^+^, CD5^+^, CD27^+^, and CD10^+^ cells by magnetic bead separation. Using this protocol, we were able to consistently obtain 70–80% pure “untouched” CD19^+^CD20^+^CD10^–^CD27^–^ B cells not bound by mAbs that might affect their responses (Additional file 2: Figure S1).

### Emab anti-CD22 does not affect the expression of BCR-inducible genes and genes associated with TLR signaling

In initial experiments, we used CD20^+^CD10^–^CD27^–^ enriched B cells to test the effects of Emab on responses to BCR and/or TLR7 stimulation. After enrichment, we preincubated cells for 1 hour at 37 °C with Emab, hIgG1 isotype control, or R10 medium and then stimulated them with the TLR7 agonist R848, F(ab′)2 anti-human IgM, or a combination of R848 and anti-IgM for 12 hours. Using quantitative RT-PCR, we measured the relative expression of multiple genes known to be induced downstream of BCR signaling (*C-MYC*, *BCL*_*XL*_, *TP53*) or associated with TLR7 signaling (*TLR7*, *TLR9*, *MyD88*, *IRF7*, *UNC93B*, *IRAK4*, *TRAF6*). Emab had no significant effect on the expression of any of these genes (Additional file 3: Figure S2 and data not shown).

### Emab modestly enhances B-cell proliferation

**Fig. 2:**
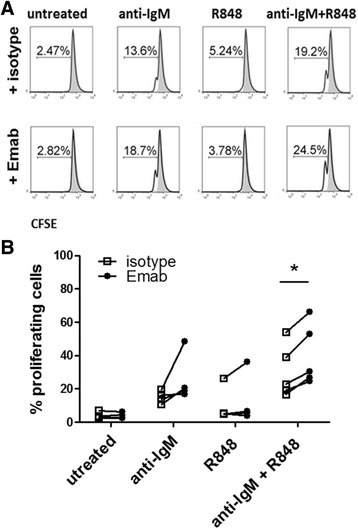
Effect of Emab on cell proliferation. Tonsillar CD10^–^CD27^–^ B cells were negatively selected using magnetic bead cell enrichment and then stimulated with TLR7 agonist R848 and/or F(ab′)2 anti-human IgM with or without Emab or a human IgG control. Cells were labeled with CFSE and cultured for 3 days with different stimuli, and levels of cell proliferation were measured based on CFSE dilution. Gates depict CFSE^lo^ (proliferating) cells. **a** Flow cytometry data from one representative experiment. **b** Cumulative data from five independent experiments using CD10^–^CD27^–^ enriched B cells from different donors. *p < 0.05 by two-way ANOVA analysis with Bonferroni post tests. *Emab* epratuzumab

To further study the effects of CD22 crosslinking by Emab on B-cell responses, we tested its effects on cell survival and cell proliferation. CD10^–^CD27^–^ enriched B cells were labeled with CFSE and stimulated with F(ab′)2 anti-human IgM, the TLR7 ligand R848, or a combination of F(ab′)2 anti-human IgM and R848. Cells were cultured for 3 days and then analyzed for cell survival and proliferation by flow cytometry. Results from five independent experiments demonstrated a small but consistent enhancement of B-cell proliferation in response to anti-IgM plus R848 in the presence of Emab (Fig. 2a, b); Emab showed no effect on cell survival in culture (data not shown). Because B-cell differentiation into effector cells usually requires cell cycle exit, these data raised the possibility that Emab might affect other B-cell functions such as their ability to produce cytokines and differentiate into Ab-producing cells.

### Emab differentially regulates BCR and TLR7-induced cytokine production

**Fig. 3:**
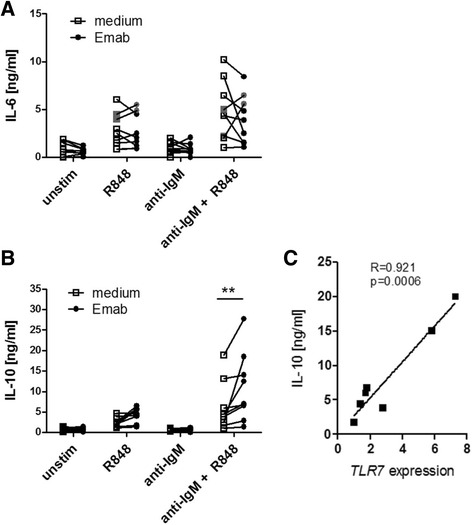
Effects of Emab on IL-6 and IL-10 cytokine production. Tonsillar CD10^–^CD27^–^ B cells were negatively selected using magnetic bead cell enrichment and then stimulated with R848 and/or F(ab′)2 anti-human IgM with or without Emab or a human IgG control. **a, b** Cells were treated with different stimuli and cultured for 3 days, and production of IL-6 and IL-10 in cell supernatants was quantified by ELISA. Cumulative data from nine independent experiments using CD10^–^CD27^–^ B cells from different donors. Experiments with increased IL-6 production in the presence of Emab are shown (*gray*) (**a**). **c** Correlation between *TLR7* mRNA expression in donor CD10^–^CD27^–^ B cells at the time of activation and the levels of IL-10 produced 3 days after anti-IgM/R848 stimulation. **p < 0.01 by two-way ANOVA analysis with Bonferroni post tests. *Emab* epratuzumab, *IL* interleukin, *TLR7* Toll-like receptor 7

To test the effects of Emab on human BCR and TLR7-induced cytokine production, we measured the level of cytokine/chemokine production in B cells cultured with or without Emab. In initial studies using cytokine arrays, we found that R848 or a combination of F(ab′)2 anti-human IgM plus R848 stimulation of CD10^–^CD27^–^ tonsillar B cells induced the production of several cytokines and chemokines, of which the most striking were IL-6 and IL-10. In a subsequent set of experiments, we isolated CD10^–^CD27^–^ tonsillar B cells from individual donors and measured the production of IL-6 and IL-10 after 3 days of cell culture with or without Emab or a human IgG control by ELISA (Fig. 3a). In seven out of nine independent experiments, the production of IL-6 in response to R848 and/or anti-IgM plus R848 was reduced in the presence of Emab. However, two experiments showed the opposite effect (i.e., increased induction of IL-6).

Notably, the induction of IL-10 in response to anti-IgM/TLR7 stimulation was significantly increased in the presence of Emab (Fig. 3b). Although the production of IL-10 varied between donors, interestingly this variation was principally due to donor variation in the expression of the TLR7 receptor (Fig. 3c). Priming of CD10^–^CD27^–^ enriched B cells with IFN-α led to a 3-fold to 4-fold increase in the expression of *TLR7* and resulted in increased production of IL-10 after IgM/TLR7 stimulation, which in turn was further increased by Emab (Additional file 4: Figure S3A and Fig. 3b). Together, these data show that Emab has differential effects on cytokine production after BCR/TLR7 stimulation, by decreasing the production of the pro-inflammatory cytokine IL-6, but promoting the immunoregulatory cytokine IL-10. The observed heterogeneity of the responses to BCR/TLR7 engagement largely reflects differences in the expression of *TLR7*.

### Emab selectively inhibits the expression of *PRDM1*, the gene encoding Blimp1

**Fig. 4:**
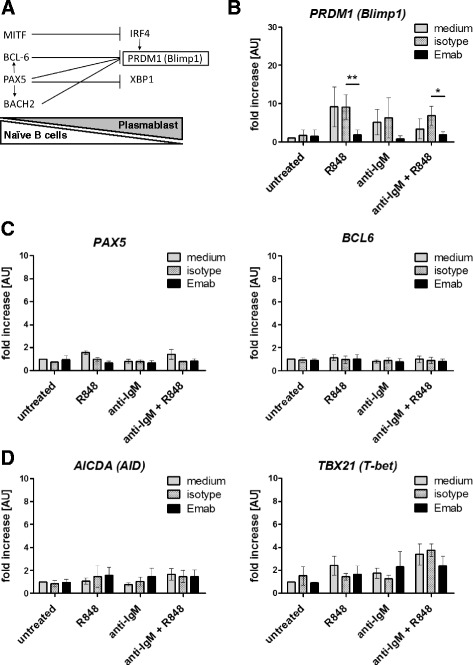
Emab selectively inhibits *PRDM1* mRNA expression in CD10^–^CD27^–^ B cells. **a** Network of transcription factors involved in B-cell differentiation into Ab-producing cells. **b**–**d** Tonsillar CD10^–^CD27^–^ B cells were negatively selected using magnetic bead cell separation and then stimulated with R848 (TLR7 agonist) and/or F(ab′)2 anti-human IgM with or without Emab or a human IgG control. RNA was isolated 12 hours after stimulation and expression of different genes was quantified by RT-PCR. *p < 0.05, **p < 0.01 as determined by two-way ANOVA with Bonferroni post test. *Emab* epratuzumab

To further determine the effects of Emab on B-cell activation and differentiation, we examined the expression of genes known to regulate B-cell differentiation from naïve into Ab-producing cells. As shown in Fig. 4a, B-cell differentiation is regulated by a complex regulatory network, consisting of transcription factors that regulate the transcriptional activation (or repression) of one another as well as other key genes involved in B-cell differentiation [62]. Initially, we examined CD10^–^CD27^–^ enriched B cells, which were preincubated with Emab, hIgG1 isotype control, or medium and then stimulated with R848, F(ab′)_2_ anti-IgM, or a combination of R848 and anti-IgM. These stimuli had no effect on the expression of *MITF*, *BACH2*, *BCL6*, *PAX5*, *IRF4*, or *XBP1* either alone or with Emab (Fig. 4c and data not shown). However, stimulation with R848, anti-IgM, or a combination of the two induced a 5-fold to 15-fold increase in the expression of *PRDM1*, the gene encoding B-lymphocyte-induced maturation protein 1 (Blimp1). Emab dramatically inhibited *PRDM1* mRNA levels, leading to an almost complete suppression of the induction of this gene after stimulation (Fig. 4b). Although we observed some variation in the increase of *PRDM1* levels, the inhibitory effect of Emab was consistently seen in six independent experiments. Remarkably, this effect appeared to be quite selective because Emab showed no significant effect on the expression of other genes involved in B-cell differentiation and immunoglobulin class-switching, including *AICDA* (encoding activation-induced deaminase) or *TBX21* (encoding T-box transcription factor, T-bet) (Fig. 4d). Thus, we conclude that signaling downstream of CD22 induced by Emab selectively inhibits *PRDM1* expression in response to TLR7 and BCR stimulation. Because Blimp1 is a key transcription factor, required by B cells to mature into Ab-producing cells, we propose that Emab might also prevent B-cell differentiation into (auto-)Ab-producing cells.

### Emab inhibits the activation and differentiation of a subset of B cells: CD27^–^IgD^–^ (DN memory) B cells that are highly responsive to TLR7 stimulation

Although CD10^–^CD27^–^ B cells are mostly thought to consist of IgD^+^ naïve B cells, recent reports have identified a population CD27^–^IgD^–^ cells that have the properties of memory B cells [4, 5, 7]. These CD27^–^IgD^–^ DN memory B cells are found in the peripheral blood and tonsils of healthy donors, but their frequency is significantly increased in patients with SLE [5]. Furthermore, an expansion of activated (CD95^+^) DN memory cells was found to correlate with SLE disease activity [4, 5]. DN memory B cells are also highly responsive to stimulation through TLR9 by CpG [4]. This led us to hypothesize that IgD^–^cells within the CD10^–^CD27^–^ population might be a subset that responds to TLR7 stimulation by upregulating *PRDM1*.

**Fig. 5:**
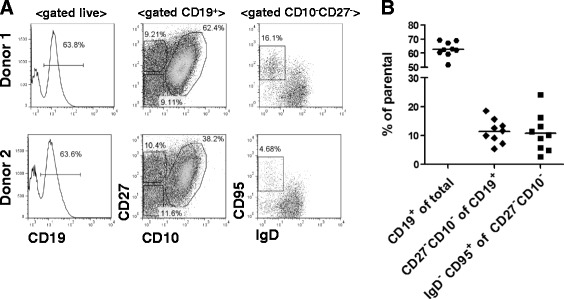
Characterization of CD27^–^IgD^–^ (DN memory) cells within tonsillar CD10^–^CD27^–^ population. **a** Representative results from flow analysis of tonsils from two individual donors. Cells were stained with a combination of mAbs against CD19, CD27, CD10, IgD, and CD95 surface markers and used to determine the frequencies of IgD^–^CD95^+^ cells within the CD19^+^CD10^–^CD27^–^ cell fraction. **b** Frequencies of different cell subsets in tonsils from nine individual donors, showing significant variation in the frequencies of DN cells

To address this question, we first assessed whether the tonsillar CD10^–^CD27^–^ B-cell subset contained DN memory B cells using a combination of CD19, CD27, CD10, IgD, and CD95 surface markers. As expected, we found that the majority of CD10^–^CD27^–^ cells expressed IgD; however, a population of IgD^–^ B cells including IgD^–^CD95^+^ (activated DN memory) cells was also present in this fraction (Fig. 5a). Analysis of tonsillar B cells from nine different donors showed that IgD^–^CD95^+^ B cells represented 2.5–24% of the CD19^+^CD10^–^CD27^–^ population (Fig. 5a, b). Thus, we concluded that CD27^–^IgD^–^ (DN memory) B cells (including CD95^+^, activated DN cells) are found in the tonsillar CD10^–^CD27^–^ fraction and might contribute to CD19^+^CD10^–^CD27^–^ B-cell responses to TLR7.

**Fig. 6:**
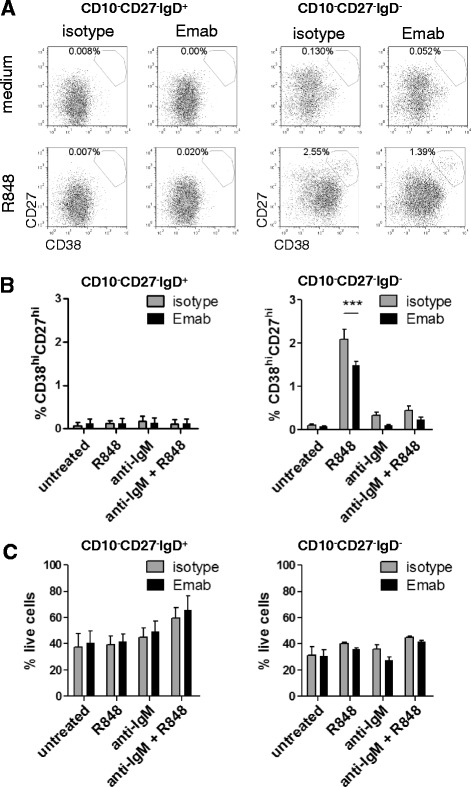
Emab inhibits the activation CD27^–^CD10^–^IgD^–^ (DN) B cells in response to TLR7 stimulation. CD10^–^CD27^–^IgD^+^ (naïve) and CD10^–^CD27^–^IgD^–^ (DN memory) B-cell subsets were sorted and left untreated or stimulated with R848, anti-human F(ab′)2 IgM, or a combination of R848 plus anti-IgM in the presence of Emab or isotype control Ab. Cells were cultured for 5 days and then cell viability and surface expression of CD38 and CD27 and cell frequencies of live cells were analyzed by flow cytometry. **a** Flow data show results from one representative experiment. Gates depict the frequencies of CD27^hi^CD38^hi^ (activated) cells in each subset (**b, c**) Cumulative data from five experiments using tonsils from different donors showing the percentage of CD27^hi^CD38^hi^ of gated live cells (**b**) or the frequencies of live cells in culture (**c**). Tonsils used in these experiments were preanalyzed and selected to contain >10% CD95^+^IgD^–^ or CD27^–^CD10^–^ cells. ***p < 0.001, as determined by two-way ANOVA with Bonferroni post test. *Emab* epratuzumab

To further test the effects of anti-IgM and TLR7 stimulation on DN memory B cells and determine whether or not Emab might affect their differentiation, we isolated highly purified CD19^+^CD10^–^CD27^–^IgD^–^ (DN memory) and CD19^+^CD10^–^CD27^–^IgD^+^ (naïve) B cells using cell sorting (detailed sorting strategy and post-sort cell analysis are presented in Additional file 5: Figure S4) and then stimulated the cells with R848, F(ab′)2 anti-IgM, or a combination of R848 and anti-IgM in the presence of Emab or isotype control. Five days later, we analyzed the cells for expression of CD27 and CD38 and for survival. As Fig. 6a shows, CD10^–^CD27^–^IgD^–^ (DN memory) cells but not CD10^–^CD27^–^IgD^+^ (naïve) B cells responded to stimulation through TLR7, as evidenced by the appearance of CD38^hi^CD27^hi^ activated cells. Cumulative data from five independent experiments showed that the TLR7-mediated activation of DN memory cells was significantly reduced in the presence of Emab (Fig. 6b). However, Emab did not affect cell survival of either DN memory or naïve B cells (Fig. 6c).

**Fig. 7:**
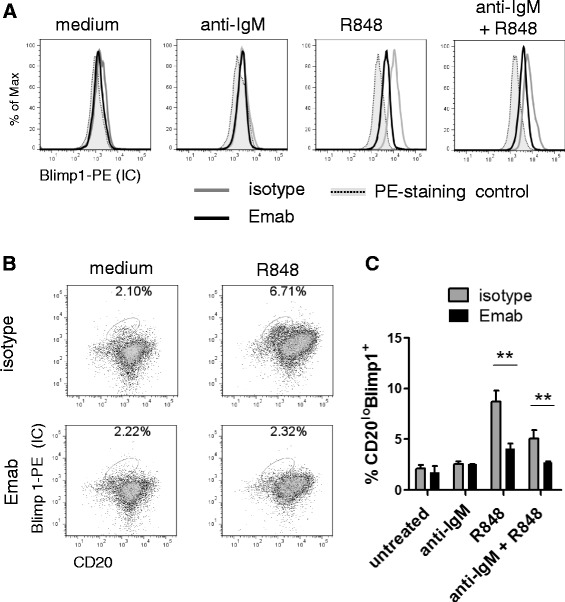
Emab inhibits the generation of Blimp1^+^ cells. Sorted CD19^+^CD10^–^CD27^–^IgD^–^ tonsillar B cells were left untreated or stimulated with R848, anti-human F(ab′)_2_ IgM or a combination of R848 plus anti-IgM in the presence of Emab or isotype control Ab. **a** Representative flow cytometry data, showing intracellular Blimp1 expression within CD10^–^CD27^–^IgD^–^ B cells after 3 days of cell culture in the presence of Emab (*black line*) or an isotype-matched control Ab (*gray line*). PE-conjugated rat IgG2a was used as a staining control (*dotted tinted line*). **b** Representative flow cytometry data showing CD20 and intracellular Blimp1 expression within CD10^–^CD27^–^IgD^–^ cells after 3 days of cell culture. Gates depict frequencies of CD20^lo^Blimp1^+^ cells. **c** Summary results from three independent experiments showing the percentage of CD20^lo^Blimp1^+^ from total live cells in culture after cell stimulation in the presence or absence of Emab. **p < 0.01, as determined by unpaired Student’s *t* test. *Emab* epratuzumab

Consistent with Emab inhibition of the upregulation of PRDM1 mRNA levels in response to anti-IgM/TLR7 stimulation, the levels of Blimp1 protein within the CD10^–^CD27^–^IgD^–^ (DN memory) subset were significantly lower in the presence of Emab (Fig. 7). As expected, stimulation of cells with R848 alone or anti-IgM plus R848 led to a significant upregulation of the Blimp1 levels (MFI for the R848-treated samples = 11,866.7 ± 2106.1), whereas the Blimp1 levels in the Emab-pretreated samples were only modestly increased (MFI for the R848-treated samples = 4773.3 ± 253.2) and significantly lower compared to the isotype-treated samples (Fig. 7a).

We found that DN memory cells also differentiated into CD20^lo^Blimp1^+^ cells upon stimulation with either R848 alone or in combination with anti-IgM. These CD20^lo^Blimp1^+^ cells also expressed high levels of CD38 and CD27, but did not express CD138 (data not shown), suggesting that they most probably represent a population of plasmablasts or preplasma cells.

The frequencies of CD20^lo^Blimp1^+^ were markedly reduced in the presence of Emab (Fig. 7b, c), further suggesting that Emab might suppress their differentiation.

Together, these data demonstrate that tonsillar CD27^–^IgD^–^ (DN memory) B cells found within the CD10^–^CD27^–^ cell fraction are highly responsive to TLR7 signals and, upon stimulation, can differentiate into plasmablast/preplasma cells. Consistent with the effects of inhibiting the expression of *PRDM1*, Blimp1 levels and Blimp1^+^ cells were significantly reduced in the presence of Emab.

### Emab affects the survival and differentiation of CD10^+^CD27^–/+^ cells

**Fig. 8:**
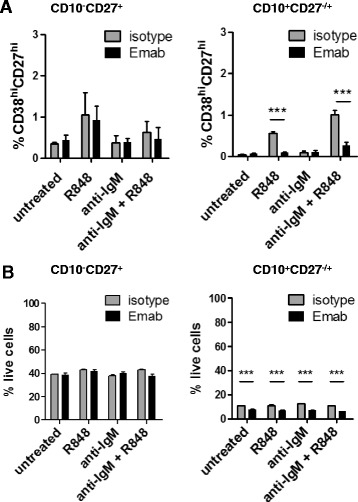
Effects of Emab on CD10^–^CD27^+^ and CD10^+^CD27^–/+^ cell activation and cell survival. Sorted B-cell subsets were left untreated or stimulated with R848 anti-human F(ab′)_2_ IgM or a combination of R848 plus anti-IgM in the presence of Emab or isotype control Ab. Cells were cultured for 5 days and surface expression of CD38 and CD27 (**a**) and frequencies of live cells in culture (**b**) were analyzed by flow cytometry. ***p < 0.001, as determined by two-way ANOVA with Bonferroni post test. *Emab* epratuzumab

To further explore the effects of Emab on different B-cell populations in tonsils, we purified CD10^–^CD27^+^ (memory) and CD10^+^CD27^–/+^ (pre-GC/CG/plasma) cells and, as in the earlier described experiments, stimulated them with R848, F(ab′)_2_ anti-hIgM, or a combination of R848 plus anti-IgM in the presence of Emab or isotype control. Cells were analyzed for the appearance of CD38^hi^CD27^hi^ activated cells and cell survival after 5 days of in-vitro culture. We found that Emab did not affect the responses of CD10^–^CD27^+^ cells, but did significantly inhibit the activation/differentiation of CD10^+^CD27^–/+^ cells in response to R848, or a combination of R848 and anti-IgM, as measured by a greater than 2-fold reduction in the frequencies of CD38^hi^CD27^hi^ cells (Fig. 8a). Analysis of cell viability showed a significant reduction in the percentage of live cells within the CD10^+^CD27^–/+^ population in the presence of Emab, with or without stimulation. This effect of Emab seems specific to this particular cell population, because the survival of CD10^–^CD27^+^ (Fig. 8a), CD10^–^CD27^–^IgD^–^ (DN memory), or CD10^–^CD27^–^IgD^+^ (naïve) B cells (Fig. 6c) was unaffected by Emab. Because the CD10^+^CD27^–/+^ subset represents a heterogeneous population of B cells, further studies are needed to determine what cells within this mixed population are affected most by Emab. Together, these results demonstrate that, depending on the B-cell developmental/maturation state, CD22 crosslinking by Emab can differentially affect B-cell survival and activation.

## Discussion

A number of recent studies have demonstrated that TLR signaling in B cells plays an important role in modulation of B-cell effector functions such as cytokine production and ability to produce auto-Abs [13]. Activation of B cells by TLR7 in particular has been proposed to play an important role in SLE [17, 20, 63]. Because of the significance of TLR7 in the pathogenesis of SLE, we assessed how targeting CD22 with Emab affected B-cell responses after TLR7 and/or BCR stimulation. We used human tonsillar B cells, which provided the means to investigate the responses of B cells from different developmental/activation stages.

In our initial studies, we used tonsillar CD20^+^CD10^–^CD27^–^ enriched B cells, which in many respects resemble CD27^–^ naïve blood B cells [64]. Because Emab has previously been shown to modulate signaling downstream of the BCR [48, 49, 57], we investigated whether Emab might also affect the expression of genes known to be activated through the BCR. While BCR or TLR7 stimulation or a combination of the two stimuli induced upregulation of a number of genes, including *cMYC* and *BCL*_*X*L_, Emab did not affect their expression. Similarly, Emab showed no significant effect on the expression of *TLR7* and *TLR9* or of *MyD88* and *IRF7* genes that encode proteins downstream of TLR7 signaling. In another set of experiments, naïve tonsillar B cells were incubated with IFN-α or a combination of IFN-α and anti-IgM. While this stimulation led to a significant upregulation of multiple genes, and in particular those involved in TLR signaling, the expression of these genes was not affected by Emab (data not shown).

However, TLR7/BCR-driven IL-6 and IL-10 cytokine production by CD20^+^CD10^–^CD27^–^ B cells were differentially modulated by Emab with a significant increase in IL-10 production and an overall inhibition of IL-6. Analysis of IL-6 and IL-10 transcripts 6, 12, or 24 hours after TLR7/BCR stimulation showed no significant effect of Emab on gene expression (data not shown); IL-10 mRNA expression was only weakly increased after TLR7/BCR stimulation and again was not affected by Emab. Thus, the exact mechanism for Emab-induced modulation of cytokine production remains to be determined. Recently, Fleischer et al. [56] analyzed the effects of Emab on the cytokine production by purified total blood B cells from healthy donors and SLE patients. They found that Emab significantly decreased the expression of TNF-α and IL-6 in response to anti-BCR and/or anti-BCR/CpG stimulation. Although anti-BCR/CpG stimulation also induced significant increase of IL-10 production, the authors found that IL-10 levels were not affected by Emab.

In contrast, we found that Emab significantly enhanced IL-10 production by naïve tonsillar B cells in response to anti-IgM/TLR7 stimulation. Interestingly, the capacity of B cells to produce IL-10 correlated positively with the expression levels of *TLR7* at the time of stimulation. In this regard, we found that prestimulation of the cells with IFN-α, a known inducer of TLR7 [26], was able to boost IL-10 production, which was further enhanced in the presence of Emab. The disparity between our results and those of Fleisher et al*.* [56] may be due to different sources of B cells being tested, use of a TLR7 vs a TLR9 agonist, and/or the levels of TLR9 or TLR7 in the B cells that were examined. We observed that IL-10 production was largely dependent on TLR7 activation by R848 and less dependent on BCR activation, a similar conclusion reached by Fleischer et al*.* although that they used a TLR9 ligand. In summary, in the majority of our experiments, Emab displayed a differential effect on cytokine production by suppressing IL-6 and inducing IL-10.

The effects of Emab on cytokine production may contribute to the understanding of the mode of action of Emab in vivo, particularly in autoimmune diseases, where B-cell-produced cytokines are believed to play an important role in driving or suppressing the disease [1]. For example, IL-6 has been implicated in B-cell differentiation and Ab production [65, 66], and is also known to cooperate with IL-21 to promote the differentiation of CD4^+^ T-follicular helper cells [67–70]. Furthermore, increased IL-6 levels have been reported in SLE patients with active disease and more recently IL-6 has been identified as a major genetic risk factor for SLE [71]. Emab-mediated IL-6-inhibition could possibly suppress inflammation associated with SLE. IL-10, on the contrary, is known to be produced by regulatory B cells [72] and has been proposed to suppress effector Th1 and Th17 cell responses [73–75]. Recent studies have suggested that IL-10 production by B cells might be defective in SLE patients [74, 76]. Based on our findings, CD22 engagement by Emab may very well help to reprogram B cells in SLE patients to restore IL-10 production.

Further studies are required to determine how CD22 signaling promotes IL-10 production by TLR7-driven B cells. Liu et al*.* [77] have recently shown that activation of STAT3 and ERK is required for TLR-induced IL-10 production by human B cells. The authors also found that IFN-α enhanced TLR7/8-induced but not TLR9-induced IL-10 production. Although it is well known that TLR stimulation elicits IL-10 production, to our knowledge this study is the first to show a role for CD22 in promoting IFN-α/TLR7-induced IL-10 production. Whereas CD22 is often described as a negative regulator of BCR signaling, it should be noted that previous studies have shown that CD22 associates with a number of signaling molecules, such as Syk, PI-3 kinase, Grb2, and phospholipase-Cγ2 [78–80], and that direct CD22 engagement can induce activation of ERK2 [81]. In line with this finding, we have found that Emab induces increased ERK phosphorylation in human B cells [82]. In light of Liu et al.’s findings [77], this might provide a mechanistic explanation of how CD22 crosslinking by Emab promotes IL-10 production.

Interestingly, Emab also modestly increases B-cell proliferation in response to BCR/TLR7 stimulation. This effect of Emab might be selective to the CD27^–^CD10^–^ (naïve and DN) cells and also dependent on the particular signals used to activate the cells. In contrast to CD27^–^ (naïve/DN) B cells, Emab did not affect cell proliferation of CD27^+^ (classical memory) cells; however, the rates of cell proliferation were variable between different donors (data not shown). Previous studies have shown that Emab can inhibit the proliferation of CD27^–^ and CD27^+^ blood B cells in response to IL-2, IL-10, F(ab′)_2_, and/or CD40L and CpG [54]. Thus, the effect of Emab on cell proliferation among various B-cell subsets needs further investigation. In the context of BCR/TLR7 stimulation of the CD27^–^CD10^–^ subset, the small increase of cell proliferation may be related to our finding that Emab inhibits B-cell differentiation. Previous studies have shown that B cells exit the cell cycle once they start to differentiate into Ab-producing plasma cells and a change in the expression of several transcription factors (e.g., a decrease of BCL6, PAX5, and c-Myc levels and an increase of Blimp1 expression) has been implicated in the transition/differentiation of naïve B cells into plasma cells [62]. In CD10^–^CD27^–^ B-cell cultures, we found that Emab dramatically inhibited the levels of *PRDM1*, the gene encoding Blimp1, which was induced in response to TLR7 and/or BCR/TLR7 stimulation. This effect of Emab was highly specific to *PRDM1* because this Ab did not affect the expression of other genes involved in B-cell differentiation or immunoglobulin class-switching, including *MITF*, *BACH2*, *BCL6*, *PAX5*, *IRF4*, *XBP1*, *AICDA*, or *TBX21*. We did see a trend toward reduction in the increase *TBX21* in response to anti-IgM/TLR7 in the presence of Emab, but the combined results from different experiments did not show statistical significance.

The responses of human B-cell subsets to TLR7 or anti-BCR/TLR7 stimulation have not been studied in detail. Recently, Simchoni et al. [28] showed that TLR7 stimulation expands IgM^+^CD27^+^ memory B cells and promotes the generation of CD27^hi^ B cells. In the current study, by comparing four different B-cell populations based on the appearance of CD38^hi^CD27^hi^ cells, we discovered that the CD10^–^CD27^–^IgD^–^ subset was the most responsive to TLR7 stimulation compared to the other B-cell subsets. Notably, the generation of CD38^hi^CD27^hi^ cells was inhibited in the presence of Emab. A portion of CD10^+^CD27^+^ and CD10^+^CD27^–/+^ B cells also differentiated into CD38^hi^CD27^hi^ cells, but their frequencies were lower compared to those generated by CD10^–^CD27^–^IgD^–^ B cells. Emab clearly decreased CD38^hi^CD27^hi^ cell frequencies, particularly within the CD10^+^CD27^–/+^ cells. CD10^–^CD27^–^IgD^+^ (naïve) B cells did not generate CD38^hi^CD27^hi^ cells in response to TLR7 stimulation. Of note, there were no significant differences in TLR7 expression between CD10^–^CD27^–^IgD^–^ and CD10^–^CD27^–^IgD^+^ B cells, suggesting that their differential responsiveness to TLR7 ligation cannot be simply attributed to increased TLR7 levels. Furthermore, a comparison between these two cell populations showed no differences in their CD22 expression and/or their ability to internalize Emab upon CD22 binding (NV Giltiay, unpublished data). Data from mouse and human studies have indicated that TLR7 can promote the production of auto-Abs [13, 28]; because Blimp1 is required by B cells to mature into Ab-producing cells, we propose that Emab-mediated inhibition of Blimp1 may reduce Ab/auto-Ab production. While the mechanisms for Emab-induced inhibition of B-cell differentiation need further elucidation, our data suggest that one therapeutic effect of Emab may be via inhibiting the expression of *PRDM1* (Blimp1). In this respect, it is highly relevant that there is elevated expression of Blimp1 in SLE patients and this is correlated with increases in plasma cells, auto-Abs, and disease activity [83].

Interestingly, Emab also affected cell survival, although this effect was only evident within the (pre-GC/GC) CD10^+^CD27^+/–^ cell population. A possible explanation for these data is that cells within this mixed cell population might be more sensitive to CD22-induced apoptosis. While apoptosis induction is not considered the primary mode of action of Emab, previous in-vitro data have demonstrated that crosslinking of CD22 by mAbs including Emab induces apoptosis in human lymphoma cells [84, 85]. Recently, Macauley et al*.* [43] demonstrated that changes in the glycosylation patterns due to altered enzyme activity in the GC leads to “unmasking” of CD22 binding site on GC B cells, relative to naïve and memory B cells. Such unmasking could very well alter the effects of CD22 binding by Emab. It should be noted that the effects of Emab on GC cell survival in culture were independent of BCR/TLR7 stimulation. These data, however, may be relevant to the clinical effects of Emab in patients with NHL, because GC B cells are considered a major source (i.e., cell of origin) for lymphoma cells [46, 86].

Despite initial promising data in phase II clinical studies [52, 53], results reported recently from a phase III clinical trial in SLE patients with moderate to severe disease showed that Emab failed to reach the primary clinical endpoint [87]. It has been suggested that a high placebo response and early rescue of nonresponders with increased doses of glucocorticoids might have confounded the data from the trial [87, 88]. Although disappointing, these results reflect to a large extent the complexity and diversity of the pathology of SLE and suggest that perhaps some, but not all, SLE patients would benefit from Emab therapy. In our study, we have found that Emab affects the production of cytokines in response to BCR/TLR7 stimulation, by skewing B cells to produce immunoregulatory cytokines such as IL-10. Thus, we can predict that targeting CD22 with Emab might be able to restore IL-10 production by CD27^–^CD24^hi^CD38^hi^ transitional B cells in SLE patients. Transitional B cells are expanded in some SLE patients and our data showed that these cells express relatively high levels of CD22, suggesting that this cell population might be a good target for Emab therapy.

Our study identified CD10^–^CD27^–^IgD^–^ as another important cell population, whose responses to BCR/TLR7 stimulation were affected by Emab. We believe this cell population closely resembles the DN memory B cells found in the blood. We found a significant variation in the frequency of this B-cell population in tonsils, which we think might be reflective of environmental factors, such as recent viral exposure. An increased frequency of DN memory B cells has been described previously in SLE patients [4, 5]. Although there has been no direct evidence for their contribution to disease pathology, DN memory B cells have been linked to SLE autoimmunity [4, 5]. For example, a significant portion of DN memory cells found in SLE patients produce VH4-34-encoded 9G4 Abs, known to be a source of SLE-associated autoreactivity [89]. Frequencies of DN memory B cells were found to be associated with higher disease activity, history of nephritis, and presence of auto-Abs [4, 5]. The cellular origin of DN memory B cells remains somewhat elusive; because these cells express switched isotypes, yet at the same time show a reduced rate of somatic hypermutations compared to post-switched memory B cells, it has been proposed that they might be of extra-GC origin [5]. Recent studies have shown that a substantial fraction of Ab-secreting cell clones found during SLE flares contained auto-Abs without (or with very few) mutations, consistent with differentiation outside GCs [90].

Whether TLR-mediated activation of DN B cells might contribute to the generation of Ab-secreting clones has not been established. One study has shown that DN memory B cells are also highly responsive to stimulation through TLR9 by CpG [4]. Here, we reported that these cells are also highly responsive to stimulation through TLR7 by R848 or a combination of anti-IgM and TLR7. Importantly, we demonstrated a new role for CD22 in regulating the activation of these cells and their differentiation into CD27^hi^CD38^hi^Blimp1^+^ plasmablasts. In this study, we did not address in detail whether DN cells produce cytokines. In a limited set of experiments, we found that DN cells produced lower levels of IL-6 and IL-10 in response to TLR7 and or BCR/TLR7 stimulation, compared to naïve B cells; however, this needs to be investigated further. Recent reports describe an increase in DN memory B cells in RA patients and older people [91, 92]. Although the link between DN B cells and SLE disease activity seems well established, we have found a significant variation of DN memory B-cell frequencies in SLE patients, even those with active disease (NV Giltiay, unpublished data). In light of these findings, one can predict that patients with high frequencies of DN memory B cells might be better candidates for CD22 targeting by Emab.The fact that Emab blocks B cell differentiation in a distinct subset of memory B cells is also consistent with our studies in mice showing that CD22 is required for normal memory B cell formation [93].﻿

Recent data suggest that Blimp1 levels positively correlate with the levels of pathogenic auto-Abs in SLE patients and can be used as a potential biomarker for monitoring disease activity [83]. Although results from the EMBODY1 and EMBODY2 trials showed no significant reduction of the total IgG levels and/or anti-DNA titers in Emab-treated patients [87], it would be of interest in the future to test whether decreases in Blimp1 levels and auto-Ab titers are associated with responses to Emab in vivo.

## Conclusions

Together, our data show that binding of CD22 by Emab can both regulate cytokine expression and block Blimp1-dependent B-cell differentiation in response to BCR/TLR7 stimulation. The effects of Emab depend on the stage of B-cell development or activation. A better understanding of the differential effects of Emab on different B-cell populations, including its effects on cell survival and cell activation, would provide important information on how best to use Emab in a clinical setting. We found that Emab inhibits the activation of CD27^–^IgD^–^ (DN) cells which are highly responsive to stimulation via TLR7 and which are known to be expanded in some but not all SLE patients. The therapeutic effect of Emab in SLE might be via modulating the production of pro-inflammatory and anti-inflammatory cytokines by B cells, or by inhibiting the survival and/or differentiation of specific B-cell populations. Because Blimp1 is required by B cells to mature into Ab-producing cells, CD22-mediated inhibition of Blimp1 may also reduce auto-Ab production. Intriguingly, given the failure of Emab to demonstrate efficacy in a general SLE patient population, levels of Blimp1 expression could possibly be used as a biomarker to predict clinical responses to Emab in SLE and in patients with other autoimmune diseases.

## Additional files

Additional file 1: Table S1. Presenting the primer sequences used for RT-PCR. (PDF 116 kb)

Additional file 2: Figure S1. Showing negative selection for CD10^–^CD27^–^ B-cell enrichment. (**A**) Schematic representation of the selection process, which involves depletion of CD2^+^ cells by rosetting, followed by depletion of CD3^+^, CD5^+^, CD10^+^, and CD27^+^ cells using magnetic bead separation. (**B**) Representative flow data showing frequencies of CD20^+^ (B cells) and frequencies of different B-cell populations after the enrichment. (PDF 138 kb)

Additional file 3: Figure S2. Showing that Emab does not affect the expression of BCR inducible genes and genes associated with TLR signaling. Tonsillar CD10^–^CD27^–^ B cells were negatively selected using magnetic bead cell separation and then stimulated with R848 (TLR7 agonist) and/or anti-human F(ab′)_2_ IgM with or without Emab or a human IgG control. RNA was isolated 12 hours after stimulation and expression of different genes was quantified by RT-PCR. Graphs show combined data of three independent experiments, presented as mean ± SD. (PDF 41 kb)

Additional file 4: Figure S3. Showing that IFN-α priming increases TLR7 expression and promotes IL-10 production, which is further enhanced in the presence of Emab. (**A**) Tonsillar CD10^–^CD27^–^ B cells were stimulated with IFN-α (100 U/ml) for 3–12 hours. Increase of *TLR7* levels presented as fold increase relative to unstimulated cells at 3 hours. (**B**) Cells were left untreated or IFN-α-primed for 6 hours, and then stimulated with R848 and/or F(ab′)_2_ anti-human IgM with or without Emab or a human IgG control. Graphs show the levels of IL-10 production after 3 days of cell culture. Data shown are representative of three independent experiments with similar results. (PDF 27 kb)

Additional file 5: Figure S4. Showing the sorting strategy for isolation of CD10^–^CD27^–^IgD^–^ and CD10^–^CD27^–^IgD^+^ cells. Tonsillar CD19^+^ B cells were enriched by rosetting and stained with fluorescently labeled mAbs: anti-CD3, CD10, CD27, and IgD Abs. CD10^–^CD27^–^ cells were separated based on their IgD expression and sorted into CD10^–^CD27^–^ IgD^–^ or CD10^–^CD27^–^ IgD^+^ populations using an Aria II high-speed sorter. Post-sort analysis shows the phenotype and purity of each of cell population. (PDF 116 kb)

## Abbreviations

Ab: Antibody
Auto-Ab: Autoantibody
BCR: B-cell receptor
Blimp1: B-lymphocyte-induced maturation protein 1
DN: Double negative
Emab: Epratuzumab
GC: Germinal center
IFN-α: Interferon alpha
IL: Interleukin
NHL: Non-Hodgkin’s lymphoma
PCR: Polymerase chain reaction
pSS: Primary Sjögren’s syndrome
RA: Rheumatoid arthritis
SLE: Systemic lupus erythematosus
TLR7: Toll-like receptor 7
