# Supplementary material for: Targeting CD22 with the monoclonal antibody epratuzumab modulates human B-cell maturation and cytokine production in response to Toll-like receptor 7 (TLR7) and B-cell receptor (BCR) signaling
Source: Arthritis Res Ther. 2017 May 15;19:91. doi: 10.1186/s13075-017-1284-2 (PMC5433084; doi:10.1186/s13075-017-1284-2)
Supplement: Supplementary file 1 — Presenting the primer sequences used for RT-PCR. (PDF 116 kb) [file 13075_2017_1284_MOESM1_ESM.pdf]

Table 1
Primer sequences used for RT-PCR

Gene	Forward	Reverse
AICDA	CAATAAGAACGGCTGCCACG	CAGGAGGTGAACCAGGTGAC
BCL6	CTGCAGATGGAGCATGTTGT	TCTTCACGAGGAGGCTTGAT
BCLXL	GAATGACCACCTAGAGCCTTGG	TGTTCCCATAGAGTTCCACAAAAG
CMYC	AAGAGGGTCAAGTTGGACAGTTGC	TTTCGGTTGTTGCTGATCTGTCT
IL-6	AGTGAGGAACAAGCCAGAGC	TCAGGGGTGGTTATTGCATC
IL-10	GGTGATGCCCCAAGCTGA	TCCCCCAGGGAGTTCACA
IRF7	TGGTCCTGGTGAAGCTGGAA	GATGTCGTCATAGAGGCTGTTGG
MyD88	GACCCCTGGTGCAAGTACC	AGTAGCTTACAACGCATGACAG
PAX5	TCCCTCATCTTCTCCAAAAGCA	GGAAATGCGCCGTTTGTAAA
PRDM1	CGAAATGCCCTTCTACCCTG	GCGTTCAAGTAAGCGTAGGAGT
TBX21	GGACTGAGATTGCCCCCATC	GCTGTCACCACTGGAAGGAT
TLR7	CGAACACCACGAACCTCACC	CCCAGTGGAATAGGTACACAGTT
TLR9	GAAGGGACCTCGAGTGTGAA	CTGGAGCTCACAGGGTAGGA
18S	GAGGGAGCCTGAGAAACGG	GTCGGGAGTGGGTAATTTGC
