# Supplementary figures and images for: Targeting CD22 with the monoclonal antibody epratuzumab modulates human B-cell maturation and cytokine production in response to Toll-like receptor 7 (TLR7) and B-cell receptor (BCR) signaling
Source: Arthritis Res Ther. 2017 May 15;19:91. doi: 10.1186/s13075-017-1284-2 (PMC5433084; doi:10.1186/s13075-017-1284-2)

**Fig. S1**

**A**

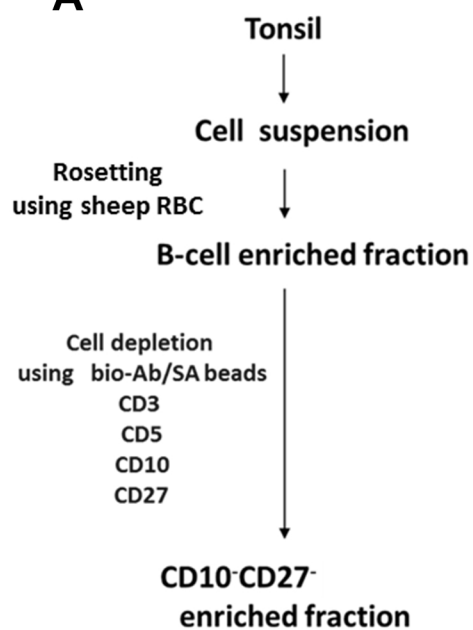

# B

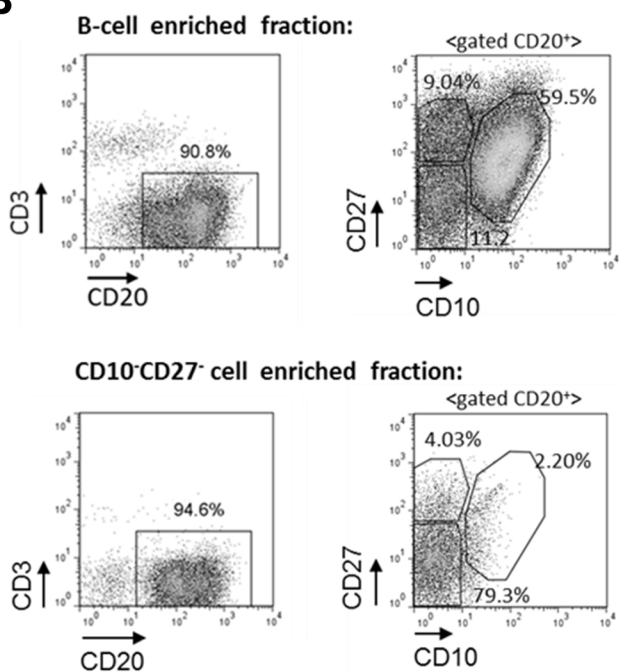

Supplement: Supplementary file 2 — Showing negative selection for CD10–CD27– B-cell enrichment. (A) Schematic representation of the selection process, which involves depletion of CD2+ cells by rosetting, followed by depletion of CD3+, CD5+, CD10+, and CD27+ cells using magnetic bead separation. (B) Representative flow data showing frequencies of CD20+ (B cells) and frequencies of different B-cell populations after the enrichment. (PDF 138 kb) [file 13075_2017_1284_MOESM2_ESM.pdf]

**Fig. S2**

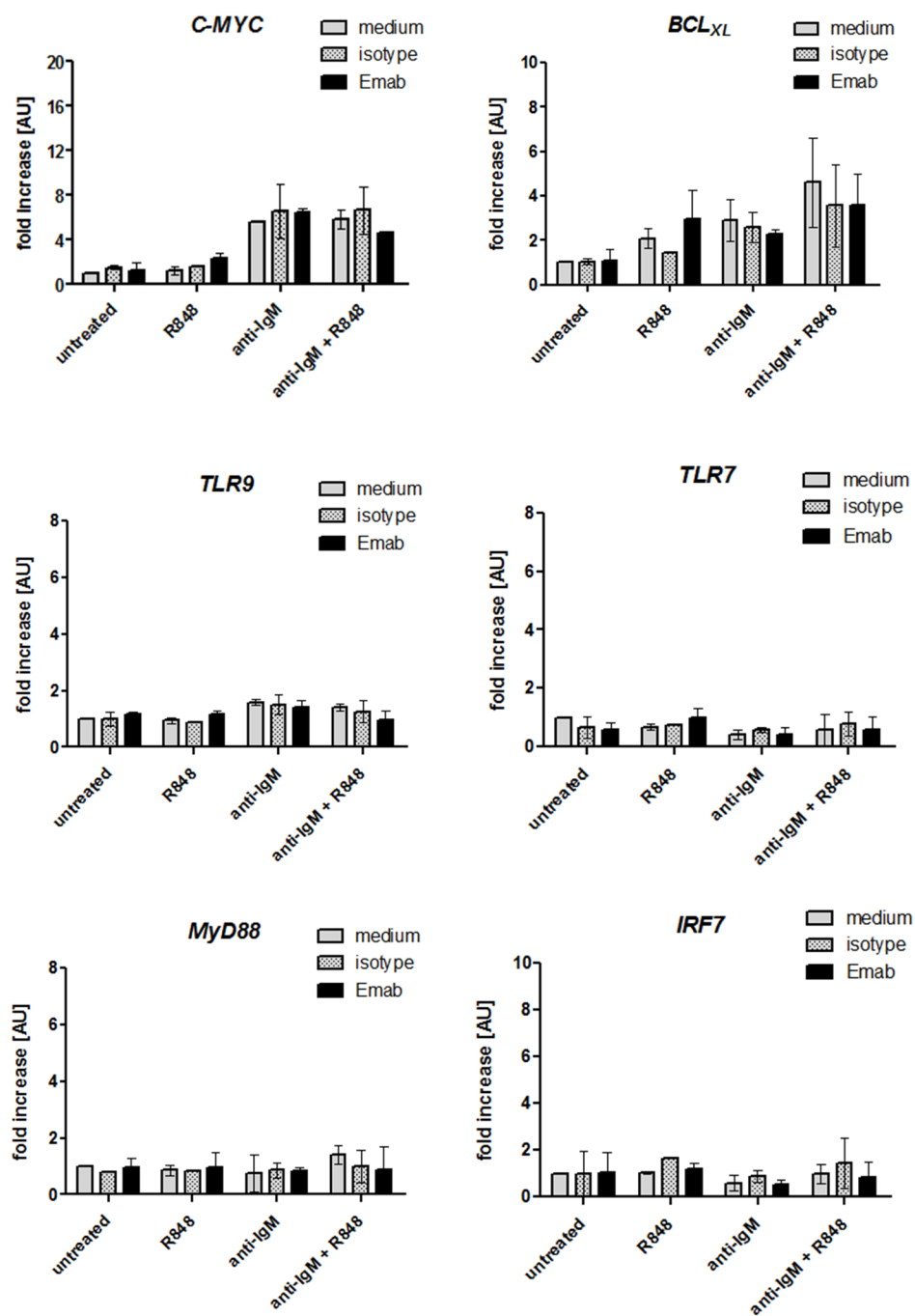

Supplement: Supplementary file 3 — Showing that Emab does not affect the expression of BCR inducible genes and genes associated with TLR signaling. Tonsillar CD10–CD27– B cells were negatively selected using magnetic bead cell separation and then stimulated with R848 (TLR7 agonist) and/or anti-human F(ab′)2 IgM with or without Emab or a human IgG control. RNA was isolated 12 hours after stimulation and expression of different genes was quantified by RT-PCR. Graphs show combined data of three independent experiments, presented as mean ± SD. (PDF 41 kb) [file 13075_2017_1284_MOESM3_ESM.pdf]

**Fig. S3**

**A**

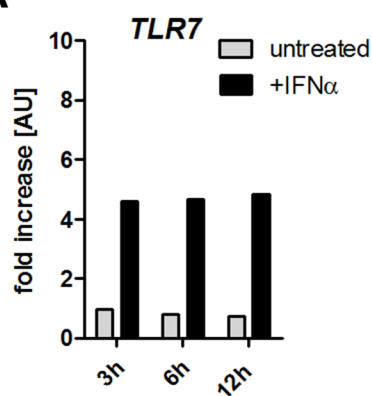

**B**

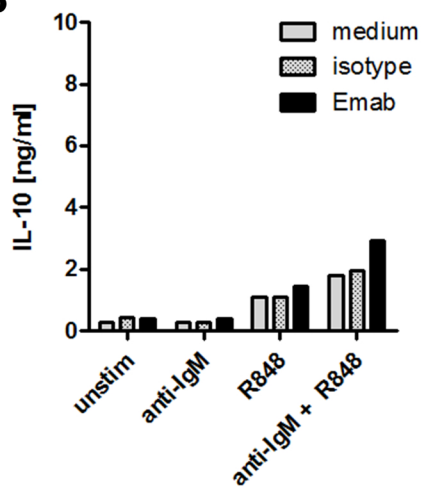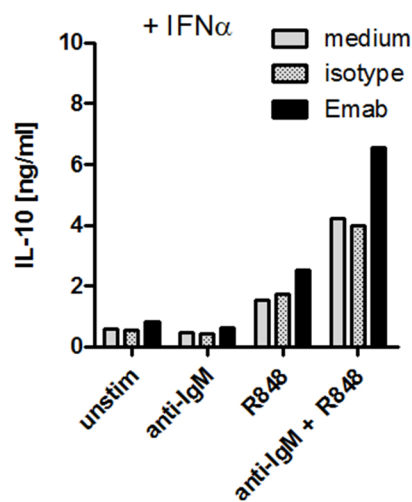

Supplement: Supplementary file 4 — Showing that IFN-α priming increases TLR7 expression and promotes IL-10 production, which is further enhanced in the presence of Emab. (A) Tonsillar CD10–CD27– B cells were stimulated with IFN-α (100 U/ml) for 3–12 hours. Increase of TLR7 levels presented as fold increase relative to unstimulated cells at 3 hours. (B) Cells were left untreated or IFN-α-primed for 6 hours, and then stimulated with R848 and/or F(ab′)2 anti-human IgM with or without Emab or a human IgG control. Graphs show the levels of IL-10 production after 3 days of cell culture. Data shown are representative of three independent experiments with similar results. (PDF 27 kb) [file 13075_2017_1284_MOESM4_ESM.pdf]

Fig. S4

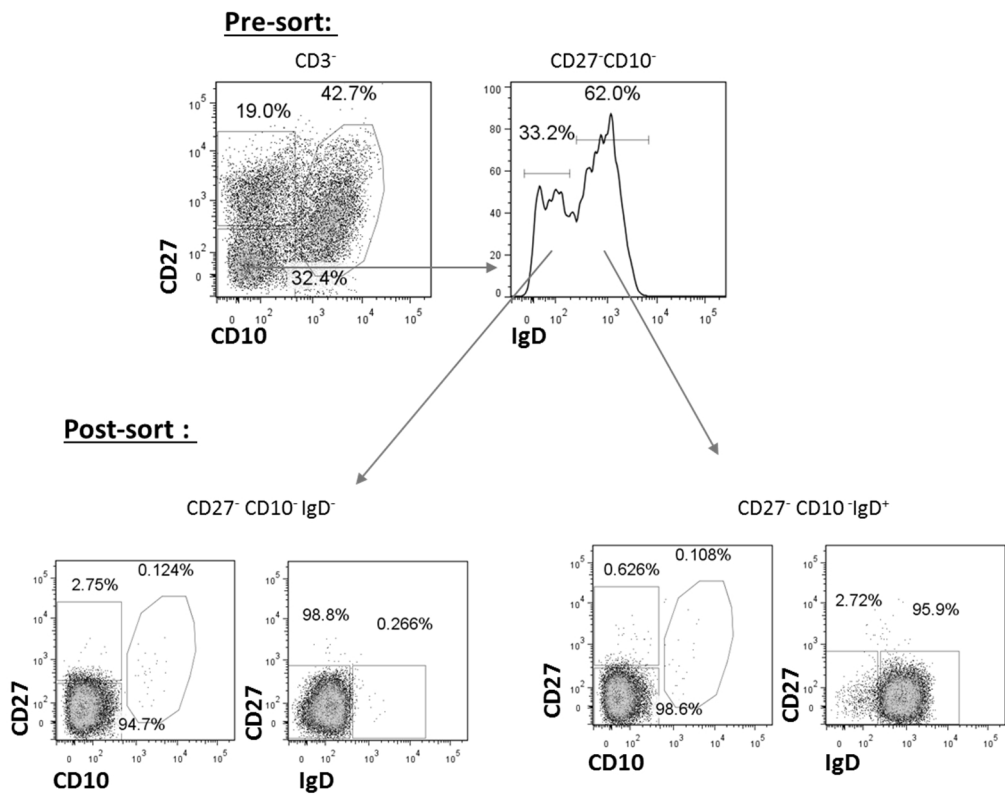

Supplement: Supplementary file 5 — Showing the sorting strategy for isolation of CD10–CD27–IgD– and CD10–CD27–IgD+ cells. Tonsillar CD19+ B cells were enriched by rosetting and stained with fluorescently labeled mAbs: anti-CD3, CD10, CD27, and IgD Abs. CD10–CD27– cells were separated based on their IgD expression and sorted into CD10–CD27– IgD– or CD10–CD27– IgD+ populations using an Aria II high-speed sorter. Post-sort analysis shows the phenotype and purity of each of cell population. (PDF 116 kb) [file 13075_2017_1284_MOESM5_ESM.pdf]
